# Attentional Reorientation and Inhibition Adjustment in a Verbal Stroop Task: A Lifespan Approach to Interference and Sequential Congruency Effect

**DOI:** 10.3389/fpsyg.2019.02028

**Published:** 2019-09-06

**Authors:** Eric Ménétré, Marina Laganaro

**Affiliations:** Faculty of Psychology and Educational Sciences, University of Geneva, Geneva, Switzerland

**Keywords:** attention, inhibition, interference, conflict adaptation, Stroop, sequential congruency effect, Gratton, verbal responses

## Abstract

Several parameters influence the interference effect elicited in a Stroop task, especially contextual information. Contextual effects in the Stroop paradigms are known as the *Gratton* or *Sequential congruency effect* (SCE). This research aims at isolating two processes contributing to the SCE in a Stroop paradigm, namely attentional reorientation from the color to the word and vice-versa, as well as inhibition (engagement/disengagement from one trial to the next one). To this end, in Study 1 subprocesses of the SCE were isolated. Specifically, attentional reorientation and inhibition were segregated by submitting young adults to a discrete verbal Stroop task including neutral trials. In Study 2, the same procedure was applied to 124 participants aged from 10 to 80 years old to analyze how interference, SCE, and the aforementioned decomposition of attention and inhibition change across the lifespan. In both studies, the Gratton effect was only partially replicated, while both attentional reorientation and inhibition effects were observed, supporting the idea that these two processes contribute to SCE on top of conflict monitoring and of other processes highlighted in different theories (contingency learning, feature integration, and repetition expectancy). Finally, the classical age-related evolution was replicated in Study 2 on raw interference scores, but no age effect was observed when processing speed was taken into account, nor on the isolated attentional reorientation and inhibition processes, which is in line with the hypothesis of stability of the inhibition processes over age.

## Introduction

At a first glance, the Stroop effect seems incredibly simple: incongruency between color word and color font interferes with color (font) naming. However, the Stroop task involves multiple cognitive processes whose effects can be disentangled. They include automatic word reading, color naming and inhibition, aiming at constraining the attentional focus on the relevant dimension. This definition of the task is relevant only when the current trial is taken into account. However, previous literature favors the hypothesis that the interference effect can vary depending on the context of the previous trials as investigated by the conflict adaptation literature. In the framework of the sequential congruency effect (SCE), or conflict adaptation effect paradigms, specific effects of the subprocesses were isolated, namely the activation and deactivation of inhibition resources from the previous trial to the current as well as the reorientation of the attentional focus from the word to the color dimension and vice versa. This paper aims at investigating these two processes (inhibition and attentional reorientation) using a SCE paradigm including neutral trials (Study 1), and their evolution in relation with age using a lifespan approach in Study 2.

In the following, we will review the Stroop, the SCE effects and their evolution across the lifespan before proposing to isolate the aforementioned subprocesses of the SCE.

### The Stroop Task and the Stroop Effect

One of the main approaches to investigate inhibition is by well-known situations of interference, such as those elicited by the Stroop task (Stroop, 1935). Traditionally, the task requires participants to vocalize the printed color from which orthographic color names are presented. The interference comes from the overlays of two inconsistent semantic pieces of information, namely the color word (reading) and the color font (naming). The initial task designed by Stroop (1935) required the participant to name consecutively all the trials from the same condition printed on a card and the experimenter measured the time spent to read the entire card. It is noteworthy to mention that the paradigm has been adapted over time as the development of informatics allowed for the task to become discrete and for reaction times to be measured for each trial and for conditions to be randomized across trials.

The cognitive mechanisms behind the Stroop effect are still largely debated, and several models tried to define the interactions between word reading and color naming dimensions. Among the first interpretations of this interference effect, the “horse-race model” (Dunbar and MacLeod, 1984) suggested that color naming and word reading processes are launched simultaneously, triggered by the stimulus onset and compete only when the two processes reach the production stage. This model represented the reference until the arising of interactionist models, and particularly the model by Cohen et al. (1990). It also gained in credit after its integration in a computational modeling of the Stroop effect (Botvinick et al., 2001, 2004). Botvinick and colleagues’ model contributed to the understanding of the main processes involved in the Stroop effect, and it also confirmed the presumed localization of the Stroop effect from a neural point of view. This derived computational model based on Cohen, Dunbar, and MacClelland’s model confirmed that the anterior cingulate cortex plays a key role in conflict detection and resolution, as suggested by ERP (Liotti et al., 2000; Hanslmayr et al., 2008; Szűcs et al., 2009), fMRI (e.g., Peterson et al., 2002; Derrfuss et al., 2005), and PET studies (e.g., Bench et al., 1993; Carter et al., 1995).

Besides trying to explain the underpinning of the Stroop effect in cognitive or brain models, researchers also tried to test the conditions of the Stroop effect by designing innovative paradigms. This apparently simple task was then derived in a variety of ways (Macleod, 1991), among which the reverse Stroop task (in which the subject has to read the word and ignore the color font) (e.g., Stroop, 1935; Abramczyk et al., 1983; Dunbar and MacLeod, 1984), the semantic Stroop task (obtained by varying the semantic closeness of the distractor from the target) (Klien, 1964), or the auditory Stroop task (modulating the verbal information and the pitch) (e.g., Hamers and Lambert, 1972; Green and Barber, 1981). Authors also manipulated the response modality which is known to play a role in the magnitude of the interference effect (White, 1969; Keele, 1972; Macleod, 1991). At a single trial level, oral responses seem to increase the interference of the Stroop effect and have no or less impact on the facilitation effect, when compared to manual responses (Redding and Gerjets, 1977; Sharma and McKenna, 1998; Lamers and Roelofs, 2011).

As presented above, embracing all the processes involved in the Stroop task is very complex. Giving an understanding including as many aspects as possible of the task was achieved by focusing particularly on two parameters which are relevant in the discrete version of the Stroop task, namely the impact of the context in which the trial occurs and the lifespan evolution of task performance. Indeed, a trial can be influenced by the properties of the previous trial, modulating the interference and facilitation effects. This contextual effect relies on the general concept of SCE, or conflict adaptation effect (Gratton et al., 1992; Botvinick et al., 2001; Mayr et al., 2003; Egner, 2007) and can be analyzed by controlling the distribution of subsequent trial types in the experiment. Additionally, interference and conflict adaptation effects tend to evolve from childhood to adulthood and through aging. These modulations need to be clarified over the entire lifespan.

### The Sequential Congruency Effect

The SCE is a widely studied effect in cognitive psychology, offering an insight into new dimensions of well-known paradigms. It is usually defined as the facilitation effect to resolve a conflict, probably attributable to a pre-activation of the conflict monitoring mechanisms (Gratton et al., 1992; Botvinick et al., 2001, 2004; Egner, 2007; Schmidt, 2013; Duthoo et al., 2014b). This effect is not limited to the Stroop task but has been applied to other paradigms such as the Flanker (e.g., Mayr et al., 2003) and the Simon task (e.g., van Gaal et al., 2010). Contextual effects were investigated according to two different approaches. First, researchers varied the proportion of trials across conditions. This manipulation constrains the attentional system to be focused on the most recurrent dimension of a stimulus, creating a cost when trials of the less frequent condition are processed (Lowe and Mitterer, 1982). For instance, if congruent trials constitute the rarest condition, latencies for these trials will increase compared to incongruent ones, reducing the interference effect. The reverse effect is obtainable by designing a task with less incongruent than congruent trials.

Second, to observe the contextual effect of the previous trials, it is possible to consider the analysis of a specific trial’s latencies depending on the condition of the previous one. This effect, also known as the Gratton effect, reflects the modulation of the attentional and executive system when evolving from a congruent to an incongruent trial and from an incongruent trial to a congruent one (Gratton et al., 1992).

To understand the mechanisms behind this effect, a comprehension of the features on which attention is focused for each condition is needed. On congruent trials, the attention is attracted to the word dimension. This statement is debated (e.g., Besner et al., 1997), but supported by some evidence. All cognitive systems tend to choose the most efficient way to process information. It has been shown that reading color words was faster than naming color patches (Cattell, 1886; Brown, 1915; Stroop, 1935). It implies that the most efficient way to process a congruent trial (e.g., the word “blue” written in blue font) would be to focus on the word instead of the color. This statement was favored by empirical evidence. The combination of word reading and color naming even speeds up latencies of congruent trials. This facilitation effect was found when congruent trials were compared to neutral words displayed in different colors (as, for instance, the word “*house*” written in blue) (van Maanen et al., 2009). It implies that a color word in a Stroop item involves the retrieval of at least the semantic information via the reading processes; even though not all word reading processes are fully implemented in the Stroop task.

Regarding incongruent trials, the attentional control is avoiding focusing on the word dimension and is centered on the color. According to Gratton et al. (1992), switching from a congruent to an incongruent trial is then more effortful than processing two consecutive incongruent trials, because in the latter context the attentional focus is already constrained on the color dimension and the conflict monitoring mechanisms are already on (Botvinick et al., 2001, 2004). In this seminal paper, the same effect was reported on congruent trials, showing a facilitation for a second congruent trial compared to a congruent trial preceded by an incongruent one. The exact cognitive mechanisms behind the Gratton effect are still intensively debated. Originally, Gratton and colleagues suggested that participants strategically expected the following trial to be from the same condition as the current one, which involved a preparation to deal with the next trial (Gratton et al., 1992). A few years later the conflict monitoring hypothesis was proposed, suggesting that the brain is equipped with a specific conflict monitoring system, which activates the resources to face a conflict (Botvinick et al., 2001, 2004). In the case of a repetition of incongruent trials, the conflict monitoring system does not need to be activated, which explains the observed facilitation of two subsequent incongruent trials. The contextual effect is no longer explained by strategic pre-orientation of attentional resources on the relevant dimension but by an automatic process located in the anterior cingulate cortex. However, this interpretation has also been questioned and alternative explanations such as the feature integration theory and contingency learning have been proposed (Duthoo et al., 2014b). It has been claimed, for instance, that the contextual effect was the result of visual features facilitation due to the repetition of the same item (Mayr et al., 2003). The experiment motivating this conclusion was centered on the Flanker task. This task contains a limited number of items, implying that the exact same item would be repeated from the previous trial to the current one multiple times during the task. In a second paradigm the effect was no longer significant since the same condition was repeated but without repetition of the exact same stimulus. Nevertheless, some studies replicated the paradigm with variable stimuli in the same condition and found a SCE (Wühr and Ansorge, 2005; Notebaert et al., 2006; Egner, 2007). A more strict interpretation of the repetition problem, called “feature integration theory” speaks for a modulation of the contextual effect if even some elements are repeated while some others are not (Hommel et al., 2004). According to the authors, each stimulus’ dimensions are binned together and stored in an “event file.” If part of the encoded features are repeated but some associated others are not, this creates an interference (Hommel, 1998).

An other issue regarding the type of stimuli included in the task has also been debated. In a Stroop task designed to elicit a SCE, only congruent and incongruent trials are usually included. However, the set of items is more limited in the congruent condition since the number of combinations of color fonts and color words is more limited in the congruent condition (both dimensions must match) as compared to incongruent condition. Therefore, the words in the congruent condition are more often associated to the correct response (i.e., the irrelevant information) which make them become more informative of the response (Mordkoff, 2012; Schmidt, 2013; Duthoo et al., 2014b). This imbalance might also contribute to the SCE, however, it probably does not explain it in its entirety either. A study using a six-colors oral Stroop task, which controlled for this bias, still found a consistent SCE (Duthoo et al., 2014a). As described above, many studies tried to identify the factors and cognitive mechanisms underlying the SCE. To further clarify the underpinning processes of the SCE, some researchers introduced neutral trials in the Flanker task (Kunde, 2003), or the Simon task (Wühr and Ansorge, 2005; Aisenberg et al., 2014). To our knowledge, Lamers and Roelofs (2011) were the only ones to design an oral and manual response Stroop paradigm including neutral trials to investigate if the Gratton effect was driven either by the reaction to the conflict, the reorientation of the attention, or by a combined effect of the two. Their results suggested that, independently of the modality of the response (verbal or manual), attention reorientation seems to be the main generator of the observed facilitation effect in the repetition of incongruent trials relative to incongruent trials preceded by another condition.

It should be noted that the conflict adaptation effect was named in different ways across the literature. It was first referred to as the Gratton effect, relatively to the seminal paper by Gratton et al. (1992), then conflict adaptation effect, and sometimes SCE. It is however not always clear whether these effects relate only to the comparison between a repetition of an incongruent trial as compared to an incongruent trial preceded by a congruent one, all the different combinations of previous and current trials, or the general contextual effects in which the current trials happen (i.e., the manipulation of the proportion of congruent and incongruent trials). In the studies presented here, since neutral trials will be added to the set of stimuli, some combinations of previous/current trials will not involve conflict, but a difference in the gradient of congruency. We will therefore use the term *sequential congruency effect* to describe all the different combinations of previous and current trials, and *Gratton effect on incongruent current trials* to name specifically the facilitation due to the repetition of incongruent trials. The facilitation due to the repetition of two congruent trials compared to a congruent trial preceded by an incongruent one will be referred to as the *Gratton effect on congruent trials*.

To sum up, the SCE results in a complexification of the more basic interference paradigm which takes into account the previous trial’s condition. Nevertheless, the effect proved its volatility since it has not been consistently replicated. Moreover, SCE and Stroop effects have been extensively investigated providing an in depth understanding of interference. Nevertheless, interference is not a static phenomenon. It is rather a dynamic process evolving throughout the human development (Comalli et al., 1962; Macleod, 1991; Li et al., 2009). However, virtually all the studies presented above recruited only young adults. A lifespan insight into the Stroop interference and SCE would therefore bring a better understanding of the ways humans process interference.

### Lifespan Perspective of the Stroop Task

As many cognitive processes, the Stroop effect follows a U-shaped curve across the lifespan (Macleod, 1991). Interference is maximal during childhood, diminishes to become minimal during adulthood, and increases again with aging (Comalli et al., 1962). In children, the Stroop effect appears within the first year of reading acquisition, is maximal at this time of life, and decreases with age. The interference U-shaped distribution was not questioned until the last two decades, specifically regarding aging. Indeed, some studies reported a deficit in inhibition for the elderly (e.g., Andrés et al., 2008), while some others show a stabilization of inhibition performances (e.g., Sebastian et al., 2013) or even an improvement (e.g., Fernandez-Duque and Black, 2006). In particular, a recent meta-analysis (Rey-Mermet and Gade, 2018) concluded that when integrating processing speed by using derivate of mixed models analyses (state-trace analyses), the specific lifespan effect of inhibition disappeared.

There is clearly a need for additional investigations on the entire lifespan, focusing on either changes from childhood to adulthood or through aging (Craik and Bialystok, 2006). Regarding the SCE evolution across the lifespan, we are not aware of any study investigating how age impacts this effect all along the lifespan spectrum. There are, however, investigations on the transition from childhood to adulthood (Waxer and Morton, 2011; Kray et al., 2012; Larson et al., 2012; Ambrosi et al., 2016; Smulders et al., 2018) and on the transition from young to older adults (Puccioni and Vallesi, 2012; Aisenberg et al., 2014; Aschenbrenner and Balota, 2015; Xiang et al., 2016). These studies suggest that children show stronger interference effects and higher error rates relative to young adults, but when combining studies from the entire lifespan spectrum, the SCE seems globally stable across development, nonetheless showing stronger magnitudes at the extremities of the lifespan continuum. Studies using electrophysiological evoked potentials also failed to observe differences in the amplitude of the evoked potentials on the N450 component between children and young adults (Larson et al., 2012), suggesting that the processes underpinning the Gratton effect are identical in children and young adults. Another ERP study on different tasks (stimulus-response compatibility, Simon task and a hybrid choice-reaction/No-Go task) found differences in the magnitude of the Gratton effect between children and young adults (Smulders et al., 2018), which disappeared after correction of processing speed differences among the age-groups. Results on aging (Puccioni and Vallesi, 2012; Aisenberg et al., 2014; Aschenbrenner and Balota, 2015; Xiang et al., 2016), suggest that the Gratton effect is preserved. This paradigm helped clarify two main hypotheses regarding decrease in cognitive performances with aging (Puccioni and Vallesi, 2012). The first one stands for a general slowing explaining the exacerbated reaction times in the elderly (Salthouse and Badcock, 1991; Salthouse, 1996), while the second suggests that the elderly suffer from a frontal lobe degeneration, altering executive performances (West, 1996; West and Bell, 1997). Since the Gratton effect remained preserved with aging, the authors suggested that their results favored the general slowing hypothesis. This conclusion was corroborated by Aisenberg et al. (2014). By increasing the inter-stimulus interval, the authors reported normalized performances regarding the Stroop effect and SCE in aging. However, it is noteworthy to emphasize that some studies failed to replicate the Gratton effect either with young adults and elderly participants (Xiang et al., 2016). Since repetitions of the same dimension were avoided in the design of the task, the authors argued that the results favored the hypothesis of the feature integration theory. The results would then be attributable to the repetition of the same dimension’s characteristic from the previous trial to the current one (Hommel, 1998; Mayr et al., 2003; Hommel et al., 2004).

To summarize, while the Stroop effect is among the strongest phenomena reported in cognitive psychology, the Gratton effect tends to be volatile, sometimes difficult to highlight and thereby rendering the interpretation of the involved cognitive processes difficult. It nevertheless emerges from the literature that the Gratton effect reflects, at least partly, the activity of the conflict monitoring mechanism (Botvinick et al., 2001, 2004; Duthoo et al., 2014b), pre-orienting the attentional focus on the relevant dimensions for the next trials. Regarding the lifespan evolution, the Stroop effect does not seem to be impacted by age, suggesting that processing speed is a much more relevant factor to explain age differences in inhibition. The SCE effect also tends to remain stable over the lifespan. Although both the SCE and lifespan approaches already represent valid methodologies to better understand the interference effect. To go further, we therefore suggest that new insights can be given through the inclusion of neutral trials to a SCE paradigm. In particular, we propose to dissociate the switching mechanisms from one dimension to another and the modulation of inhibition processes from one trial to the next over the entire lifespan, as detailed in the ensuing section.

### The Present Study: Beyond the Sequential Congruency Effect

**FIGURE 1:**
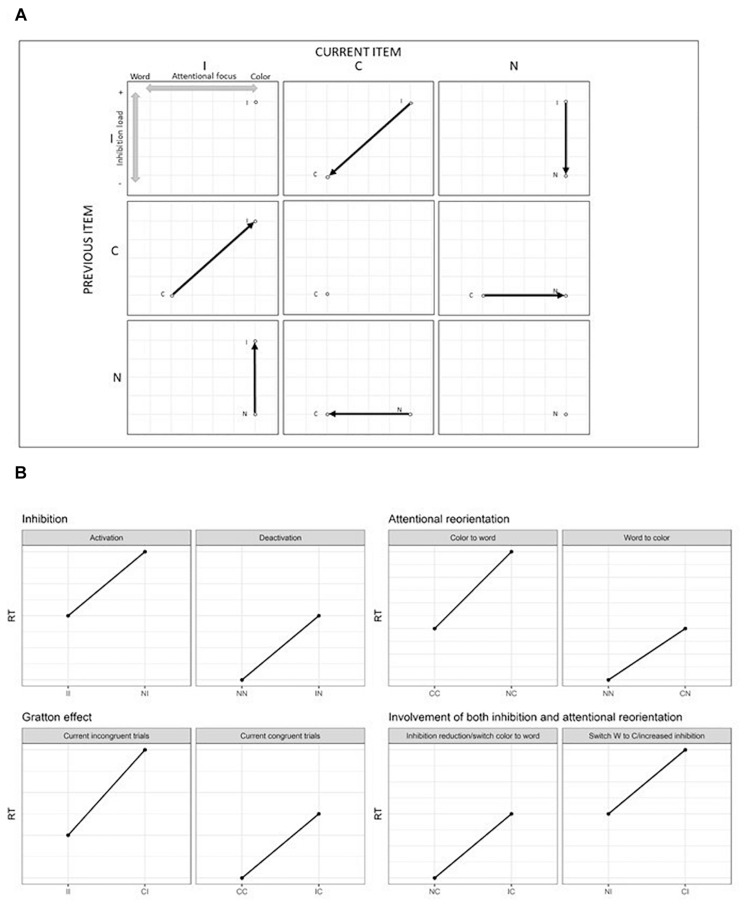
**(A)** Theoretical reorientation of attentional focus (from the color to the word and from the word to the color dimension) and activation/deactivation of the inhibition load from the previous trial to the current one. Dots represent repetition of the same condition, while vertical and horizontal displacement reflect a change on only one dimension (either attentional or inhibition), while diagonal displacements reflect a change on both dimensions simultaneously. **(B)** Expected results regarding the dissociation between inhibition and attentional processes, SCE and impact of both attention and inhibition. I, incongruent trial; C, congruent trial; N, neutral trial.

As described above, the increased reaction times resulting in an interference effect in the Stroop task encompass the SCE. In particular, the SCE can be further decomposed into the bidirectional reorientation of the attentional focus between the word or the color dimension and the engagement/disengagement of inhibition processes. To our knowledge, no study has tried to demonstrate this dissociation so far. However, some previous studies investigated the phenomenon of attentional capture by a salient stimulus close to the target followed by a reorientation of the attention from the distractor to the relevant target (Folk et al., 1992; Lamy et al., 2004; Serences et al., 2005; Chang et al., 2013). More precisely, a relatively close paradigm to the Stroop task was proposed in this domain (Serences et al., 2005; Chang et al., 2013). In these studies, the participants were asked to spot the red central letter among rows of letters displayed in different colors. The surrounding letters could be in red, in different colors, or in black. This paradigm allowed to isolate either the attentional capture effect alone or associated with the reorientation of the attention. Results suggest that attentional reorientation is a specific mechanism and the related brain regions activated involve mainly the temporo-parietal junction (although the involved brain network responsible for this reallocation of the attentional resources is still debated: Corbetta and Shulman, 2002; Geng and Vossel, 2013; Diquattro et al., 2014). Even though this task has some similarities with the Stroop task, it does not involve word processing. Moreover, in the SCE, the changes are made sequentially from the previous trial to the current one, while in the Serences et al. (2005) and Chang et al. (2013) studies, targets and distractors were presented simultaneously. To understand how the SCE was decomposed in subprocesses in the present study, Figure 1A represents the nine possible combinations of transitions from the previous trial to the current one along with changes in the attentional focus and inhibition cost in a Stroop paradigm including neutral trials. In the present study, neutral trials were sequences of symbols presented in colored ink.

Figure 1B represents the expected results according to the division of the SCE into Gratton, inhibition and attentional reorientation effects. It is expected from the predictions of the Gratton effect, that when processing a repetition of incongruent (II)^1^ trials, the second incongruent trial will show faster responses than an incongruent trial (I) preceded by a congruent trial (C), i.e., that (II < CI). A repetition of congruent trials (CC) should also be performed faster than a C trial preceded by an I (IC). Crucially, as shown in Figure 1B, an incongruent trial preceded by a neutral trial (NI) involves the engagement of inhibition load but no attentional reorientation. To isolate inhibition, an NI trial can be compared to an II trial, which involves the same processes, except for the engagement of the inhibition load. The same logic can be applied to the deactivation of the inhibition, by comparing IN trials to NN ones. To isolate the attentional reorientation processes, the cognitive cost caused by the switching from the color to the word dimension can be assessed by the comparison between NC and CC trials. The investigation of the opposite effect, namely the reorientation from the word to the color dimension, can be measured by comparing CN to NN trials. Finally, the Gratton effect implies a change in both dimensions simultaneously. It is nevertheless possible to identify other situations where both dimensions operate simultaneously. Between NC and IC, inhibition processes are deactivated while in both conditions the attention is reoriented from the color to the word dimension. In the NI – CI comparison, the attentional focus switches across dimensions and inhibition has to be activated. Therefore, the Gratton effect should be considered as the sum of the two subprocesses, since it involves a change in both dimensions.

Study 1 will focus on testing the theoretical framework described above regarding the decomposition of the SCE in attentional reorientation mechanisms and engagement or disengagement of the inhibition processes, then, Study 2 will analyze its evolution over the lifespan.

## Study 1

This study tests the SCE decomposition, inhibition, attentional reorientation effects and interactions between the two processes by adding neutral trials to the standard Stroop task on a group of young adults.

### Method

#### Participants

Twenty seven young adults (mean age = 24.4 years old, *SD*: 3; 17 women) were recruited for the purpose of this study. They were all native French-speakers, did not report any neurological, psychiatric, color vision or language impairment and received a financial compensation for their participation to the study. All of them gave a written informed consent and the local Ethics Committee approved the entire procedure (see Appendix 1 for the gender and age distribution).

#### Materials

The stimuli were those of discrete standard Stroop tasks (from Fagot et al., 2008), namely four French color words (“bleu”; “jaune”; “rouge”; “vert”, respectively: blue, yellow, red, and green) displayed in lower case, at the center of the screen in one of the four possible colors. The neutral stimuli were arrays of symbols (“++++”; “^^^^”; “ ”” ”; “^****^”) presented in one of the four colors. The stimuli were either congruent (the color word matched the color in which the word was displayed), incongruent (the color word was displayed in a different color font) or neutral (a non-verbal symbol displayed in one of the different possible color fonts).

The total number of trials was 180, equally distributed among the three conditions (60 congruent, 60 incongruent, and 60 neutral). Stimuli order was pseudo-randomized by the Mix software (Van Casteren and Davis, 2006) to avoid the repetition of the same item, and allow a repetition of the same condition for a maximum of three consecutive trials. In addition, a color presented in the previous trial (target or distractor level) was not present in the following one, to prevent visual (and verbal) repetition, according to the “feature integration theory” (Hommel et al., 2004). As described in the Introduction section, the interference effect is maximized in SCE paradigms since word-color combinations are more numerous in the incongruent condition compared to the congruent one. This effect is known as the contingency learning effect (Mordkoff, 2012), and was controlled in the present study by adding neutral trials. In a Four colors Stroop task of 180 items, each color is therefore presented 15 times per Stroop condition.

#### Procedure

The subjects sat approximately 80 cm from a 17-inches screen (refreshment rate: 50 Hz). The experiment was performed on the E-prime software (E-Studio). Oral responses were recorded by a dynamic microphone, digitally amplified and the signal was redirected to a computer. Subjects had to produce only oral responses and reaction times were obtained by marking manually the onset of the production (the delay between stimulus presentation and vocal onset) during the pre-processing stage using the Check Vocal software (Protopapas, 2007).

Each trial of the Stroop task began with a 500 ms white fixation cross on a black background, followed by a 200 ms black screen. The stimuli were then displayed on a black background for 1500 ms, followed by a variable interstimulus black screen lasting from 1000 to 1200 ms. The timing was identical for all age groups. Participants were systematically asked to name the color in which the stimuli were displayed as fast and as accurately as possible, independently of the written sequence. Before the beginning of the task, the participants were trained on 32 trials including all possible stimuli combinations, to make sure they understood the task and to avoid a novelty effect on the first trials.

#### Data Analysis

For data cleaning purposes, a trial was considered as incorrect if the subject produced the wrong color name (even if the response was corrected), or if the subject did not give any answer. Incorrect responses and latencies exceeding two standard deviations from the individual mean reaction times were excluded from the latency analysis. For the congruent, incongruent and neutral conditions, the percentage of rejected trials were respectively 3.83, 17.07, and 3.02%. See Appendix 5 for the percentage of excluded trials among SCE conditions. In addition, the first trial of each subject has not been analyzed as a SCE trial since there was no previous trial.

Analyses were performed using the R software (R Core Team, 2019). Data wrangling was mainly performed using the *dplyr* (Wickham et al., 2019) and *tidyr* (Wickham and Henry, 2019) packages, while statistical analyses were computed with the base package, *lmerTest* (Kuznetsova et al., 2017) and *Lme4* (Bates et al., 2017) using the mixed models *lmer* function. Errors were analyzed by generalized mixed models using the *glmer* function.

Since contrasts were explored by turning over the intercept variable of the model to target all relevant comparisons, the resultant multi-testing bias was corrected using the Bonferroni method (Bonferroni, 1936). Therefore, the significance threshold was divided by the number of necessary models.

### Results

**FIGURE 2:**
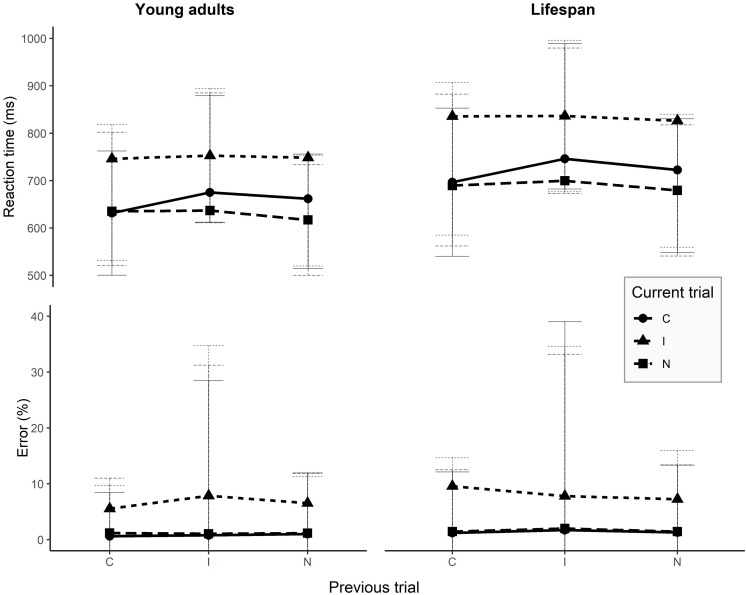
Reaction times and error rates for SCE conditions on young adults and over the entire lifespan. Error bars represent one standard deviation above and under the mean. I, incongruent; C, congruent; N, neutral.

In the Stroop task, overall production latencies were the fastest for neutral trials, and latencies were faster on congruent trials than on incongruent trials. Table 1 displays the mean reaction times for the current condition and for the previous trial condition. When considering the previous trial’s condition, an incongruent previous trial causes larger interference on the processing of the next trial than any other condition, while the congruent condition seems to be facilitatory for the next trial. Regarding accuracy, the best performance was observed in the congruent condition, while the incongruent condition generated the highest error rate. A previous incongruent trial tends to lead to higher error rates on the current trial, while a congruent previous trial minimizes the chances to commit errors as compared to the other two conditions. SCE latencies and error rates are presented in Figure 2.

**TABLE 1:** Mean reaction times and accuracy rate per condition, separately for the current and the previous trial.

	**Current trial condition**		**Previous trial condition (mean of all current conditions)**	
	
	**Mean (*SD*)**	**Accuracy (*SD*)**	**Mean (*SD*)**	**Accuracy (*SD***)
Congruent	645.08 (130.88)	99.2% (0.09)	657.16 (135.95)	98% (0.14)
Incongruent	732.53 (125.61)	93.15% (0.25)	689.24 (142.62)	96.17% (0.19)
Neutral	620.43 (114.23)	98.77% (0.11)	677.25 (143.07)	96.99% (0.17)

Results of the Stroop and SCE were included in a linear mixed model. Model selection was performed by loading the random part of the model with all relevant variables as random slopes and intercepts, and reducing this random part until the model converged. Then, the most complex random structure able to converge was adopted (regarding model selection, see Zuur et al., 2009). More precisely, in a first model, all fixed factors were added as a random slope (Barr et al., 2013; Matuschek et al., 2017). Since the model did not converge, interactions between factors was removed at first and then the less relevant factors were removed hierarchically from the model. After several attempts, only the current trial was supported as random slope. Regarding random intercepts, the participants (Subject) and Items variables were retained. Regarding fixed factors, the model included the previous and current condition, the stimuli presentation order as well as the interaction between the previous and current condition to account for an eventual learning or fatigue effect occurring during the task (complete model detailed in Table 2A).

**TABLE 2:** **(A)** Results of the mixed model analysis for the young adults group.

**(A) Model: lmer(log(RT) ∼ Previous Condition ^∗^ Current Condition + stim. Presentation order + (1 + current condition | Subjects) + (1| Items), data = data, REML = FALSE)**						

		**Sum of square**	**Mean square**	**Num/Den DF**	***F***	***p***
Previous condition		0.57	0.28	2, 4246.9	16.41	*p* < 0.001^∗^
Current condition		2.99	1.49	2, 41.4	86.33	*p* < 0.001^∗^
Stim. Presentation order		0.85	0.85	1, 4354.8	49	*p* < 0.001^∗^
Previous ^∗^ Current condition		0.66	0.16	4, 4234.8	9.5	*p* < 0.001^∗^

**(B) Comparisons**		**df**	**β**	***SE***	**t**	***p***

Current trial	C – I	55.15	0.68	0.2	8.33	*p* < 0.001^∗^
	I – N	71.97	−0.16	0.01	−10.31	*p* < 0.001^∗^
	C – N	47.65	0.01	0.01	0.22	*p* = 0.828
Previous trial	C – I	4363	0.06	0.01	6.88	*p* < 0.001^∗^
	I – N	5358	−0.02	0.01	−2.56	*p* = 0.01^∗^
	C – N	4369	0.04	0.01	4.8	*p* < 0.001^∗^

*In first line the lmer R function generating the model and **(B)***post hoc* results (after Bonferroni correction, ^∗^the significance threshold equals 0.025) of the current and previous trials condition.*

The general model showed a main effect of the current condition, the previous condition, a main effect of stimuli presentation order, as well as a significant interaction between the previous and the current condition.

The Stroop effect (latencies on incongruent trials compared to congruent ones) was replicated. Moreover, latencies on incongruent trials were also significantly slower than on neutral ones. Finally, the congruent and neutral conditions did not differ significantly. The decomposition of the previous trials main effect showed that all contrasts are significant, which confirms that compared to both neutral and congruent conditions, an incongruent previous trial interferes with the processing of the following one (see Table 2B for detailed results).

**FIGURE 3:**
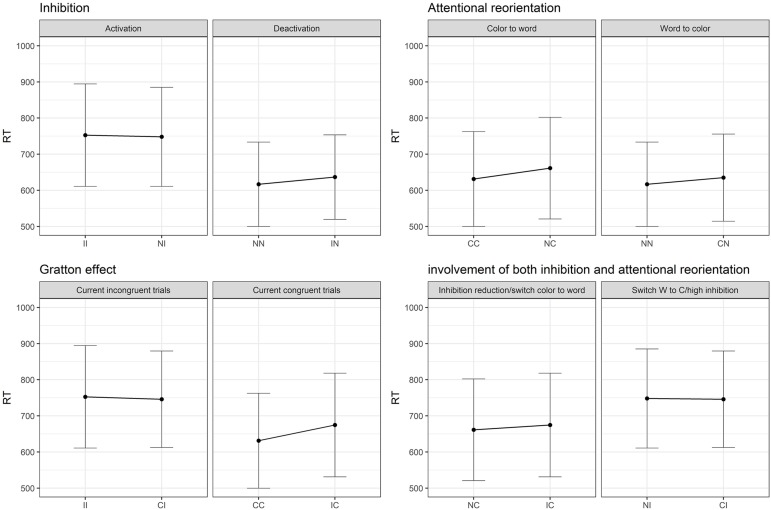
Results of the SCE decomposition in Study 1. I, incongruent trial; C, congruent trial; N, neutral trial. II = incongruent trial preceded by an incongruent trial (and so on).

Regarding the *post hoc* decomposition of the SCE conditions, six models (two per current trial condition) were necessary to estimate the results. The original data was divided in three data frames, one for each current trial condition, and one model was run for each data frame including the previous trial condition (presented in Table 3). Since there are three previous possible conditions, two models per condition were necessary. As shown in Figure 3, there is only a partial effect of the SCE, since the Gratton effect on incongruent trials was not replicated (II trials are not performed faster in comparison to CI trials). This result is in opposition to the Gratton effect on congruent trials (facilitation for CC trials compared to IC trials), which was strongly significant. Regarding the division of the SCE, concerning the attentional reorientation, both effects returned significant. Although, regarding the inhibition activation and deactivation, only the contrasts corresponding to deactivation (NN vs. IN) reached significance. As shown in Figure 3, neither of the comparisons implicating an effect of both attention and inhibition simultaneously reached significance.

**TABLE 3:** Results table containing the R commands of the mixed models analyses generating all the *post hoc* comparisons.

**Model: lmer(log(RT) ∼ Previous.trial + (1| Subject) + (1| Items), data = data.current.C/data.current.I/data.current.N, REML = FALSE)**						

**Current item**	**Comparisons**	**df**	**β**	***SE***	***t***	***p***
Congruent	CC – IC	1530.98	0.06	0.01	6.2	*p* < 0.001^∗^
	CC – NC	1522.04	0.04	0.01	4.2	*p* < 0.001^∗^
	NC – IC	1531.17	−0.02	0.01	−2.43	*p* = 0.015
Incongruent	II – NI	1531.16	−0.02	0.01	−2.42	*p* = 0.015
	II – CI	1165.98	0.17	0.01	1.86	*p* = 0.062
	NI – CI	1266.03	0.01	0.01	0.69	*p* = 0.492
Neutral	NN – CN	1500.81	−0.03	0.01	−3.37	*p* < 0.001^∗^
	NN – IN	1494.48	−0.03	0.01	−3.99	*p* < 0.001^∗^
	CN – IN	1512.85	0.01	0.01	0.7	*p* = 0.474

*To obtain these values, six models were mandatory, implying that, according to Bonferroni correction method, ^∗^the significance threshold is reduced to 0.008.*

Errors were analyzed using generalized linear mixed models according to a binomial distribution. The results (see Table 4) show a significant difference between the congruent and incongruent conditions with increased error rates in the latter, as well as between the incongruent and neutral condition, but no difference between the congruent and neutral conditions was seen.

**TABLE 4:** Results of the generalized linear mixed models estimating differences between conditions regarding the accuracy.

**Model: glmer(Accuracy ∼ Current trial condition + (1| Subjects), family = binomial, data = data)**				

**Comparison**	**β**	***SE***	**z**	***p***
C – I	−2.23	0.29	−7.66	*p* < 0.001^∗^
I – N	1.79	0.24	7.4	*p* < 0.001^∗^
C – N	−0.44	0.35	−1.24	*p* = 0.215

*Two models were necessary to perform all the comparisons, according to the Bonferroni correction, ^∗^the significance threshold equals 0.025.*

### Discussion Study 1

This first study aimed at establishing whether the SCE embedded other processes which can be isolated by adding neutral trials to a Stroop task. As highlighted in the Introduction section, two processes can be isolated: an attentional reorientation mechanism from the color to the word dimension and vice versa and the engagement/disengagement of the inhibition load. Results of Study 1 partially sustain the involvement of these processes. First, the Gratton effect on incongruent trials was not replicated, even though the same effect on congruent trials, i.e., a facilitation of CC trials relative to IC trials, was observed. As already described in the Introduction, the Gratton effect on incongruent trials is very volatile and several studies failed to replicate it (Mayr et al., 2003; Lamers and Roelofs, 2011; Kreutzfeldt et al., 2016; Xiang et al., 2016). From a cognitive point of view, the effect relies on a reduction of the interference effect by a preparedness from the attentional and executive systems to face a conflict. However, if the conflict is too strong for the current trial, the facilitation effect is reduced or suppressed. The cost of the incongruent trials may explain the absence of a Gratton effect in the present study. However, the absence of the Gratton effect may also be related to a limited number of trials (*N* = 180) or subjects (*N* = 27), or to the presence of the neutral trials themselves. It has actually been reported that increasing the stimulus set size might increase the interference effect (Gholson and Hohle, 1968; Fraisse, 1969; Macleod, 1991), but this factor is also known to reduce the SCE (Kray et al., 2012). In the present experiment, by adding neutral trials, the number of stimuli increased, reducing the possibility to anticipate the next trial’s condition (Gratton et al., 1992; Schmidt and De Houwer, 2011). Moreover, since the design of the study was done to control for the bias described in the “features integration theory” (Hommel et al., 2004), there was no repetition (on both dimensions, i.e., the color font or the color word) from the previous trial to the current one. When this effect is controlled for, a strong reduction of the SCE is observed, therefore also contributing to the absence of effect in this study. Moreover, by introducing neutral trials, the classically observed contingency learning effect was controlled for. As described in the Introduction, this effect reflects the non-conscious association between the color word and the color font as in a Stroop task including the same number of congruent and incongruent trials, the color word is significantly more often associated with the correct response. However, in the present paradigm, the effect has been counterbalanced since neutral trials were added.

It has been suggested in the literature that the Gratton effect is due exclusively to the preparedness to the conflict resolution (Botvinick et al., 1999, 2001, 2004). However, when adding neutral trials, some of the comparisons do not contain conflictual trials, and facilitation effects were also highlighted. This suggests that conflict adaptation as triggered by a preactivation of the conflict monitoring hypothesis, is a component of the SCE but it is not the only mechanism involved. Attentional reorientation and specific manipulation of the inhibition load (activation/deactivation) seems to be one of them. Here, with the isolation of further processes, a significant increase of RTs was observed for the conditions including an attentional reorientation from the color dimension to the word dimension and from the word dimension to the color one. Since only congruent and neutral trials entered this comparison, these results tend to validate that the mechanism of attentional reorientation also causes an increase of RTs independently of incongruence. Finally, the activation of the inhibition resources from the previous trial to the current one was not significant (NI as compared to II), whereas deactivation increased the latencies (IN as compared to NN). To understand this effect, we need to emphasize that the SCE involving incongruent current trials relies on a diminution of the interference. This partial effect could reflect the fact that a threshold is reached after which the SCE is not strong enough to minimize the interference.

Before any further interpretation of the results, we will investigate in Study 2 whether the same results are observed with a larger sample and how these effects evolve over the lifespan.

## Study 2

The paradigm was virtually identical to Study 1, but involving a larger sample of participants covering six age groups from school-age children to 80 year-old adults. As discussed in the Introduction, the interference effect is known to remain stable over the lifespan, except if processing speed is controlled for. We will therefore divide the classical interference index (I-C) by the neutral trials’ latencies to control for processing speed. As processing speed is neutralized in the SCE analyses (since only one dimension is manipulated independently of the other involved processes), the results of the SCE should remain globally stable with aging. This trend should also be generalizable to the other age groups (school-aged children), even though the underlying cognitive processes are probably different. Although inhibition seems to remain stable across the lifespan, the literature does not provide hints about the lifespan evolution of other executive processes such as the reorientation of the attentional focus to one specific dimension. However, under the assumption that the absence of lifespan effects is generalizable to other executive mechanisms, a lifespan evolution of attentional reorientation should not be observed either.

### Method

#### Participants

Hundred and twenty four participants, including the first 20 participants from Study 1 [aged 10–80 years-old, mean age: 39.8 years (*SD* = 24)] were recruited from six age groups (10–13, 16–18, 20–30, 40–50, 60–70, 70–80). All participants were native French-speakers, without self-reported neurological, language, color perception, or psychiatric impairment and received a financial compensation for their participation to the study. All participants signed a written consent and the entire procedure was approved by the local Ethics Committee.

#### Materials and Procedure

Materials and procedure were identical to Study 1 (see Appendix 1 for gender and age distribution among the different age groups).

#### Data Analysis

Data preprocessing followed the same procedure as described in Study 1. Four participants with mean RTs more than 2 SD away from the mean of their age-group were excluded (one from the age-group 16–18, one from the age-group 40–50, and two from the age-group 60–70). Regarding extreme reaction times, congruent, incongruent and neutral conditions respectively showed a 4.36, 16.77, and 3.3% of rejected trials. For SCE conditions over the lifespan and specifically per age groups, see Appendix 5.

To analyze whether processing speed plays indeed a role over the lifespan, in addition to the standard interference index (estimated by subtracting the averaged congruent latencies from the incongruent ones:*I–C*), a second score was computed by dividing the standard interference score by the averaged neutral trials latencies for each subject. A one-way ANOVA assessed the evolution of each of these scores across the lifespan.

The SCE and the isolation of attentional reorientation and inhibition activation or deactivation was investigated by mixed models analyses, following the same principles as analyses performed in Study 1, except for adding the age groups as a fixed effect. Error analyses followed the same logic.

### Results

#### Corrected and Uncorrected Interference Indexes Over the Lifespan

**FIGURE 4:**
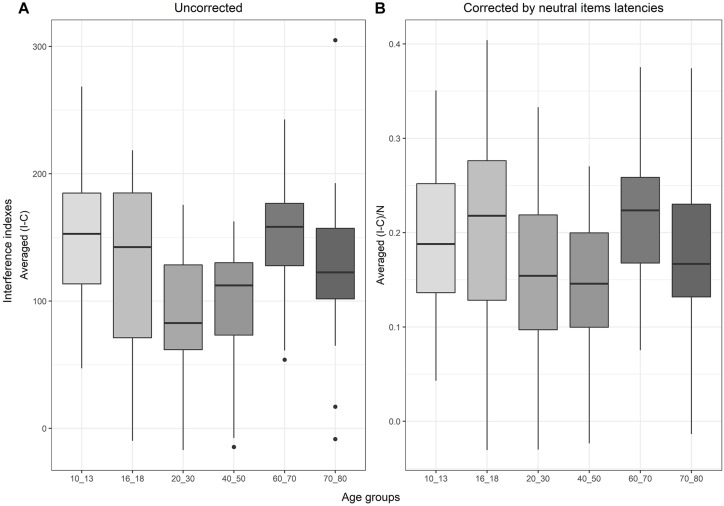
Boxplots representing, in **(A)** the evolution of the interference score (subtracting the latencies of congruent items from the incongruent ones), and in **(B)** the interference score when corrected by neutral latencies to remove processing speed from the index.

Regarding the evolution of the standard interference score (*I - C*) over the lifespan, the one-way ANOVA for uncorrected interference indexes revealed a significant main effect of age groups [*F*(5,111) = 4.07, *p* = 0.002]. According to the Tukey test and as shown in Figure 4, children aged 10–13 were significantly slower than young adults aged 20–30 [*t*(111) = 3.3, *p* = 0.02, *SE* = 18.29, β = 60.34], and than adults aged 40–50 years [t(111) = 3.27, *p* = 0.02, *SE* = 18.53, β = 60.62]. Older adults (60–70 years old) showed larger interference than younger adults [relative to 20–30 years old: *t*(111) = −3.05, *p* = 0.03, *SE* = 18.8, β = −57.4; and to 40–50: *t*(111) = −3.03, *p* = 0.03, *SE* = 19.03, β = −57.69].

With the corrected interference index(*(I–C)/N*), the analysis showed a main effect of age groups [*F*(5,111) = 2.36, *p* = 0.04], but none of the *post hoc* comparisons reached significance after correction (see Appendix 2 for detailed results).

#### Sequential Congruency Effects

Latencies and error rates of the SCE conditions are presented in Figure 2 and Appendix 3. The results of the mixed model on latencies are presented in Table 5. The final model highlighted a significant main effect of the current trial condition, a significant main effect of the previous trial condition, and a significant main effect of stimuli presentation order. Previous and current trial conditions interact with age and, crucially, a significant interaction between the previous trial condition and the current condition is observed, without a triple interaction with age.

**TABLE 5:** **(A)** Main effects and interactions of the general model, and **(B)** contrasts on current and previous trial condition.

**(A) Model: lmer(log(RT) ∼ Previous.trial^∗^Current.trial^∗^Age.groups + Presentation.order + (1 + Current.trial| Subjects) + (1| Items), data = data, REML = FALSE)**						

**Main effects and interactions**			***F***	**df**	***p*-value**	
Current trial condition			218.84	2, 41.47	*p* < 0.001^∗^	
Previous trial condition			83.28	2, 18417.78	*p* < 0.001^∗^	
Age groups			14.69	5, 116.94	*p* < 0.001^∗^	
Presentation order			8.64	1, 18890.22	*p* = 0.003^∗^	
Previous ^∗^ current trial condition			33.24	4, 18382.17	*p* = 0.001	
Current trial condition ^∗^ age-groups			2.06	10, 118.3	*p* = 0.033^∗^	
Previous items condition ^∗^ age-groups			2.93	10, 18859.53	*p* = 0.001	
Previous ^∗^ current items condition ^∗^ age groups			0.98	20, 18859.64	*p* = 0.479	

**(B)**	**Comparison**	**β**	***t***	**DF**	***SE***	***p***

Current trial	C – I	0.24	10.5	189, 24	0.02	*p* < 0.001^∗^
	C – N	0.02	1.2	149, 22	0.02	*p* = 0.23
	N – I	−0.21	−10.68	246, 06	0.02	*p* < 0.001^∗^
Previous trial	C – I	0.08	7.73	18870	0.01	*p* < 0.001
	C – N	0.05	4.83	18890	0.01	*p* < 0.001
	N – I	−0.04	−3.39	18870	0.01	*p* < 0.001

*^∗^The significance threshold was reduced to 0.008.*

Since raw reaction times were expected to evolve following a U-shaped curve, a linear model could be biased relative to the quadratic shape of the curve. To further analyze this issue, two models were generated. The first one compared age groups from children to young adults, while the second one compared the young adults to older ones. Results were globally similar for both halves of the lifespan, except for stimuli presentation order, which was no longer significant in the second part of the lifespan, as well as the interaction between the current trial and the age groups which was significant only for the second half of the lifespan (results are presented in Appendix 4).

The *post hoc* decomposition of the model presented in Table 5B highlighted a significant difference between current congruent and incongruent conditions (the standard Stroop interference effect), as well as significantly slower reaction times for the incongruent condition compared to the neutral one. Congruent and incongruent conditions were not significantly different. Regarding the previous trials condition, all three conditions differ from each other, and a previous incongruent trial alters the processing of the next trial, independently of the next trial’s condition.

**FIGURE 5:**
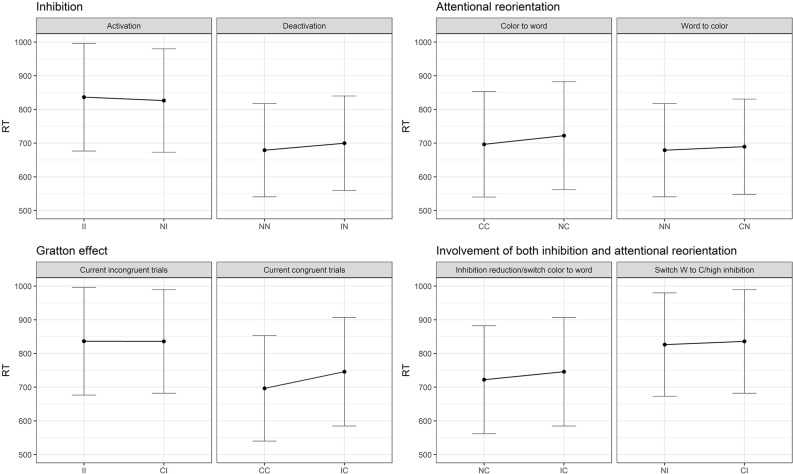
Graphical representation of the results grouped according to the dimension switching and the inhibition modulation hypotheses in Study 2.

Since the interaction between the previous and current conditions was significant as well, but not the triple interaction with age groups (see Table 5A above), contrasts were computed across all age-groups. As in Study 1, the original data frame was divided in three sets, each including only congruent, incongruent or neutral current trials conditions. A mixed model analysis was performed on each data frame with the previous trial’s condition as a fixed effect, the previous trial’s condition as random slopes, and subjects and item as random intercepts. Results are presented in Table 6 and Figure 5.

**TABLE 6:** Summary table of the mixed models used to estimate the SCE conditions.

**Model: lmer(log(RT) ∼ Previous.trial + (1| Subject) + (1| Items), data = data.current.C/data.current.I/data.current.N, REML = FALSE)**							

**Current item**	**Comparisons**	**RT(*SD*)**	**df**	**β**	***SE***	***t***	***p***
Congruent	CC – IC	−49.5(−4.8)	6587.59	0.07	0.01	13.77	*p* < 0.001^∗^
	CC – NC	−27.7(−3.9)	6495.89	0.03	<0.01	6.8	*p* < 0.001^∗^
	NC – IC	−23.7(−0.9)	6595.42	–0.04	0.01	–7.64	*p* < 0.001^∗^
Incongruent	II – NI	10(6.4)	5438.21	–0.01	0.01	–3.12	*p* < 0.001^∗^
	II – CI	0.6(6.1)	4784.87	<0.01	<0.01	0.32	*p* = 0.75
	NI – CI	−9.4(−0.3)	5364.33	–0.01	<0.01	–2.33	*p* = 0.02
Neutral	NN – CN	−10.2(−3)	6535.87	–0.01	<0.01	–3.27	*p* = 0.002^∗^
	NN – IN	−20.4(−2)	6529.69	–0.03	<0.01	–7.79	*p* < 0.001^∗^
	CN – IN	−10.2(1)	6551.47	0.02	<0.01	4.98	*p* < 0.001^∗^

*Seven models were necessary to estimate all the comparisons. After Bonferroni correction, ^∗^the significance threshold was reduced to 0.008.*

Errors were analyzed using generalized linear mixed models. The model included as fixed effects the current and previous conditions, and the Subject and Item variables as random factors. Interactions between the previous, the current trial and with the age-groups was not included in the final model since it failed to converge. Results, as presented in Table 7, suggest that all conditions were significantly different from each other.

**TABLE 7:** Summary table of the generalized linear mixed model used to appreciate the differences between the three current items conditions regarding errors.

**Model: Summary** (**glmer (TR ∼ Previous condition + Current condition + (1| Subjects) + (1| Items), data = data, family = “binomial”))**					

	**Comparison**	**β**	***z***	***SE***	***p***
Current trial	C – I	−2.29	14.33	0.16	*p* < 0.001^∗^
	C – N	1.82	14.37	0.13	*p* < 0.001^∗^
	N – I	−0.46	−2.54	0.18	*p* = 0.01^∗^

*To estimate these *post hoc*, two models were necessary, implying that ^∗^the significance threshold was divided by two and equals 0.025.*

### Discussion Study 2

This second study aimed at assessing interference and SCE as well as the specific contribution of their subprocesses over the lifespan. The present study examined effects of a bidirectional attentional reorientation and engagement or disengagement of inhibition in a larger sample, as well as their evolution over the entire lifespan.

First, the standard Stroop interference index showed a main effect of age groups and significant differences, especially between the groups at the two extremities of the lifespan and those in the middle. However, the corrected version of the interference index ((I–C)/N) did not show any significant difference across age. This finding is in line with the literature claiming that there are no influences of aging on performance when the processing speed factor is controlled for (Aisenberg et al., 2014; Rey-Mermet and Gade, 2018; Smulders et al., 2018).

Second, the results on the SCE replicated those of Study 1 with a larger sample, showing again that the Gratton effect on incongruent trials is not robust (see Discussion of Study 1). Contrariwise, clear effect of attentional reorientation in both directions (from color to word or from word to color) appeared significant. On the larger group of Study 2, both activation and deactivation of inhibition slowed down production latencies, whereas only deactivation reached significance in Study 1. Notably, the inhibition activation effect goes in the opposite direction, suggesting that, inconsistent trials seem to be more effortful when preceded by an incongruent trial (II) than when preceded by a neutral trial (NI). This favors the interpretation mentioned in the discussion of Study 1, suggesting that the SCE was not powerful enough to reduce the interference effect. This effect in contradiction with the predictions (II slower than NI) might reflect an overload in terms of cognitive control, impacting nonetheless the previous trial but there seems to be a carry-over effect of the previous trial, impacting also the successive one. This finding is in line with the literature on other tasks suggesting that there is a reset of the attentional system (Kreutzfeldt et al., 2016), requiring time before being able to process a new item. This interpretation is supported by the *post hoc* decomposition of the results showing that a previous incongruent trial increases significantly the latencies of the current one, independently of the current trial condition.

As described above, subprocesses were isolated from the SCE by adding neutral trials to a Stroop paradigm. These subprocesses are namely the disengagement of the inhibition resources and the reorientation of the attention, either from the word to the color dimension or the opposite direction. Both of the subprocesses returned significant with a larger sample. Moreover, the comparison involving both mechanisms: the transition from NC to an IC trial translating the cost due to an inhibition reduction while the attentional system is redirected to the word dimension is now significant. This new effect favors a role of the sample size in the results.

## General Discussion

The present study aimed at investigating if different processes embedded in the Stroop interference effect and more precisely the SCE could be disentangled. In particular, we tried to isolate the effect of attentional reorientation from the color word to the color font dimension and the engagement/disengagement of the inhibition resources from one trial to the next. This was achieved by adding neutral trials to a SCE in a Stroop paradigm (non-verbal signs displayed in different colors) requiring oral responses. In a first study, this dissociation was tested on a group of young adults, while in Study 2, the isolation of attentional reorientation and inhibition processes was investigated on a larger sample, covering the entire lifespan.

### Standard Stroop Interference Effect

The standard interference effect was found in young adults as well as in the other age groups. The effect was larger in the youngest and oldest groups, following a U-shaped curve over the lifespan as previously reported (Comalli et al., 1962; Macleod, 1991; Li et al., 2009). Nevertheless, processing speed seems to be responsible for a large part of the observed difference among age groups. Indeed, when interference is corrected for processing speed (by dividing the interference index by the latencies of the neutral trials), age effects disappear. This latter observation is in line with recent results on aging (Rey-Mermet and Gade, 2018). To our knowledge, the effect of processing speed on differences in interference observed in the younger age groups have not been reported so far. Since interference is known to remain stable with aging when processing speed is controlled for, it was expected that the same effect would be observable for the group of children. The involved mechanisms are not necessarily identical for the two extremities of the lifespan, even though the behavioral results are similar. Interestingly, our results confirmed that for children as well, processing speed is the major dimension responsible for the evolution of the standard Stroop interference index. This finding implies that processing speed plays a key role in the evolution of the latencies over the lifespan.

### Sequential Congruency Effect

As reviewed in the Introduction, several theoretical accounts provide an understanding of the processes underlying the SCE. In Study 1 in a sample of young adults, there was a significant Gratton effect on congruent current condition whereas the same facilitation on incongruent trial did not reach significance. Among the possible interpretations of the lack of Gratton effect on Study 1 we evoked the sample size. However, this assumption has been disproved since the results remained globally identical in Study 2 on 124 participants. The quite low number of trials (180) could have been an explanation of the results, nonetheless, a study comparing a 384 trials task with a 192 trials one, reported virtually the same results, namely a consistent SCE in the Stroop task for young adults and elderly subjects (Aschenbrenner and Balota, 2017).

Regarding the involved mechanisms, the present study supports at least a mechanism of modulation of the inhibition load, which could favor the hypothesis of conflict monitoring. Moreover, the results strongly support attentional reorientation as a component of the effect. However, despite the fact that the design of this study controlled for biases such as the contingency learning effects (Mordkoff, 2012; Duthoo et al., 2014b), features integration theory (Mayr et al., 2003; Hommel et al., 2004), and repetition expectancy (by increasing the stimulus set size) (Gratton et al., 1992), no facilitation effect was found for a repetition of incongruent trials (II) as compared to CI. These effects might have diminished the Gratton effect on incongruent current trials, explaining the non-significant result. This interpretation is corroborated by the unexpected result that a repetition of incongruent trials (II) is more interfering than NI. Nevertheless, these alternative mechanisms cannot explain the entirety of the effect, as already suggested in the literature (Egner, 2007; Schmidt, 2013; Duthoo et al., 2014b). It is therefore possible that the Gratton effect could emerge with such a paradigm only when the right balance between interference effect and SCE is found. This can be achieved by reducing the interference effect, for example by adding an asynchrony of the stimulus onset between the color word and the color font presentation. It has been suggested that the Stroop interference effect could be reduced when presenting the color word distractor 400 ms before the color to name (Glaser and Glaser, 1982; Coderre et al., 2011) or when using a single centered colored letter (Besner et al., 1997; Augustinova et al., 2010). Regarding the Gratton effect on congruent trials, our results are in line with the literature and show that CC sequences are processed faster than IC sequences, which favors the hypothesis of a carry-over effect of the interference from a previous incongruent trial to the next one. In the same way age effects no longer appeared for speed corrected interference, age did not seem to interact with the SCE. This finding suggests that there is a potential stability of the effect over the entire lifespan. It seems therefore that control adjustment processes are functional already in school-age children and are preserved during aging. There is a discrepancy in the literature regarding the evolution of the SCE over the lifespan. Since no previous study investigated the entire lifespan, results will first be confronted to the results of studies investigating the changes during development and then to those on aging. From childhood to adulthood, the SCE is present and is consistently increased in children as compared to young adults. Regarding ERP data, results tend to show a stability of the N450 between children and adults (Larson et al., 2012). The present results seem to be in opposition with these arguments, as previous literature tends to favor the hypothesis that there is an evolution of overall performances. Results on older age groups are not as consistent. On the other hand, some results on the evolution of the SCE in aging suggest that the effect is increased, at least in a Stroop task with button press responses (Aschenbrenner and Balota, 2017), and in other attention and inhibition tasks (Smulders et al., 2018). Other studies tend to show a stability of performances across ages (Aisenberg et al., 2014; Larson et al., 2016). The present results also favor a stability of performances over the lifespan. However, as neutral trials were included in the present design, we cannot exclude that the SCE relies at least partly on the ability of the participant to predict the next trial’s condition (Gratton et al., 1992; Mordkoff, 2012; Duthoo et al., 2014b). In which case results may evolve across the lifespan with a different design (with only congruent and incongruent trials). Nevertheless, such an explanation based on prediction cannot support the entirety of the SCE effects, since some contextual effects were reported on other SCE conditions.

Latencies show that elderly subjects only need more time, which may underlie the observed changes in raw performance (Aisenberg et al., 2014). This observation is in line with the general slowing hypothesis (Salthouse and Badcock, 1991; Salthouse, 1996), and is less compatible with the specific frontal lobe degeneration hypothesis leading to a reduction of executive functions, since all age groups seem to be homogeneous (West, 1996; West and Bell, 1997). However, since this conclusion relies on an absence of effect, further investigations should try to replicate these results.

### SCE Versus Standard Interference Effect

Finally, the main aim of this study was to demonstrate that the SCE allows the isolation of other embedded processes, namely the reorientation of the attentional focus from the color to the word dimension or to the opposite direction and the engagement or disengagement of the inhibition load. The results of Study 1 and Study 2 support the implication of such mechanisms on top of the Gratton effect. However, the detailed subprocesses are clearly at play when the current trial is inconsistent, whereas they are not significant or effects are in the unexpected direction on the incongruent current trials. As suggested also by the results of Lamers and Roelofs (2011), the cost of processing incongruent trials is probably too high to allow sequential switching effects to emerge on incongruent current trials, but it clearly affects negatively the processing of the following trial whichever it is (see Table 1). This might be due to a carry-over effect of the interference from the previous trial, affecting the next one. To sum up, from the moment a Stroop trial is presented among other trials, the context in which it is presented will have an impact on the intensity of the interference effect. It has been widely described that conflict monitoring is a mechanism involved in the preparedness to react to a potential conflict (Botvinick et al., 2001, 2004; Egner, 2007). The present results nevertheless suggest that attentional reorientation as well as specific adjustments of the inhibition load also play an important role in this effect.

Some limitations must be addressed in the current study. First, the number of trials (180) is not as high as in the majority of the studies. Although it has been suggested that this factor should not impact the results (Aschenbrenner and Balota, 2017), and additional studies seem necessary to understand how such effects are modulated by the number of trials. Second, by attempting to correct for a maximum of biases in the study such as the contingency learning effects (Mordkoff, 2012; Duthoo et al., 2014b) and the features integration theory (Mayr et al., 2003; Hommel et al., 2004), the number of SCE items are not perfectly balanced across all combinations of previous-current trials, which could have impacted the results. However, by adding neutral trials, the number of items increased as well, making the prediction of the next trial’s condition much more difficult.

## Conclusion

The two studies showed that the SCE can be further decomposed into attentional reorientation mechanisms (from the word to the color dimension and from the color to the word dimension) and the engagement/disengagement of the inhibition load from one trial to the next. This was achieved by including neutral trials in a SCE design of a Stroop task. The results suggest that both decomposed processes are relevant to (young) adults as well as over the entire lifespan. The identified SCE subprocesses do not change across the different age groups, as well as the standard interference effect when processing speed is controlled for. The present findings also confirm that the Gratton effect is very volatile and might be influenced by the presence of neutral trials, or other task design related effects. Finally, the present studies highlighted the importance of taking into account the attentional reorientation as much as inhibition modulation mechanisms when dealing with interference effects.

## Data Availability

The datasets generated for this study are available on request to the corresponding author.

## Ethics Statement

Human Subject Research: The studies involving human participants were reviewed and approved by the Ethics Committee of the University of Geneva. Written informed consent to participate in this study was provided by the participants’ legal guardian/next of kin.

## Author Contributions

Both authors contributed directly to the realization of the manuscript and approved it before submission. EM was involved in the data acquisition, analyses, interpretation, and manuscript writing and editing. ML supervised the work, and actively contributed to the manuscript edition and revision.

## Conflict of Interest Statement

The authors declare that the research was conducted in the absence of any commercial or financial relationships that could be construed as a potential conflict of interest.

## Footnotes

1 Hereafter, transitions are indicated as the first letter of the previous trial next to the first letter of the current trial. For example, a congruent item preceding an incongruent one will be mentioned as a CI trial.
