# Supplementary material for: Attentional Reorientation and Inhibition Adjustment in a Verbal Stroop Task: A Lifespan Approach to Interference and Sequential Congruency Effect
Source: Front Psychol. 2019 Sep 6;10:2028. doi: 10.3389/fpsyg.2019.02028 (PMC6743350; doi:10.3389/fpsyg.2019.02028)
Supplement: Supplementary file 1 [file Data_Sheet_1.docx]

APPENDICES

Appendix 1. Subjects characteristics. N is the number of subjects in the age group and age is in years.

| Age-groups | N  (N Male) | Age M (SD) |
| --- | --- | --- |
|  |  |  |
| 10-13 | 20 (12) | 11.1 (0.8) |
| 16-18 | 21 (9) | 16.6 (1) |
| 20-30 | 20 (9) | 24.9 (3.2) |
| 40-50 | 20 (8) | 45.8 (3.8) |
| 60-70 | 23 (11) | 64.3 (3.1) |
| 70-80 | 20 (5) | 73.5 (3.7) |
| All participants | 124 (54) | 39.8 (24) |

Appendix 2. Summary of the averaged reaction times of the current trial either depending on the current trial condition or on the previous trial condition over the entire lifespan.

| Condition | Current trials condition | |  | Previous trials condition | |
| --- | --- | --- | --- | --- | --- |
|  | mean RT (SD) | Accuracy % (SD) |  | Mean RT (SD) | Accuracy % (SD) |
| Congruent | 717.43 (160.11) | 98.64% (0.11) |  | 722.42 (160.96) | 96.75% (0.18) |
| Incongruent | 832.71 (156.17) | 91.98% (0.27) |  | 763.06 (164.57) | 95.65% (0.2) |
| Neutral | 691.35 (140.72) | 98.3% (0.13) |  | 744.29 (163.47) | 95.55% (0.18) |

Appendix 3. Table results describing the evolution of the interference index either corrected ((I – C)/N) or uncorrected (I – C) over the lifespan.

|  | I – C | | | | |  | (I – C)/N | | | | |
| --- | --- | --- | --- | --- | --- | --- | --- | --- | --- | --- | --- |
| Comparison | Estimate | SE | Df | t | p-value |  | Estimate | SE | Df | t | p-value |
| 10-13 – 16-18 | 24.34 | 18.29 | 111 | 1.33 | *p* = 0.767 |  | 0.01 | 0.03 | 111 | 0.28 | *p* = 0.999 |
| 10-13 – 20-30 | 60.34 | 18.29 | 111 | 3.3 | *p =* 0.016*** |  | 0.04 | 0.03 | 111 | 1.6 | *p* = 0.603 |
| 10-13 – 40-50 | 60.62 | 18.53 | 111 | 3.27 | *p =* 0.017* |  | -0.06 | 0.03 | 111 | -2.04 | *p* = 0.324 |
| 10-13 – 60-70 | 2.93 | 18.8 | 111 | 0.16 | *p = 1* |  | -0.02 | 0.03 | 111 | -0.7 | *p* = 0.984 |
| 10-13 –70-80 | 28.84 | 18.29 | 111 | 1.58 | *p* = 0.616 |  | 0.02 | 0.03 | 111 | -0.7 | *p* = 0.98 |
| 16-18 – 20-30 | 35.99 | 18.29 | 111 | 1.97 | *p* = 0.368 |  | 0.05 | 0.03 | 111 | 1.88 | *p* = 0.42 |
| 16-18 – 40-50 | 36.27 | 18.53 | 111 | 1.96 | *p* = 0.373 |  | 0.07 | 0.03 | 111 | 2.36 | *p* = 0.193 |
| 16-18 – 60-70 | -21.41 | 18.8 | 111 | -1.14 | *p* = 0.864 |  | -0.12 | 0.03 | 111 | -0.40 | *p* = 0.999 |
| 16-18 – 70-80 | 4.95 | 18.3 | 111 | 0.25 | *p =* 0.999 |  | 0.04 | 0.03 | 111 | 1.33 | *p* = 0.766 |
| 20-30 – 40-50 | 0.28 | 18.53 | 111 | 0.01 | *p = 1* |  | -0.01 | 0.03 | 111 | -0.47 | *p* = 0.997 |
| 20-30 – 60-70 | -57.4 | 18.8 | 111 | -3.05 | *p* = 0.033* |  | 0.06 | 0.03 | 111 | 2.23 | *p* = 0.231 |
| 20-30 – 70-80 | -31.5 | 18.3 | 111 | -1.72 | *p* = 0.52 |  | 0.01 | 0.03 | 111 | 0.55 | *p* = 0.994 |
| 40-50 – 60-70 | -57.68 | 19.03 | 111 | -3.03 | *p* = 0.035* |  | -0.79 | 0.03 | 111 | -2.66 | *p* = 0.09 |
| 40-50 – 70-80 | -31.78 | 18.53 | 111 | -1.71 | *p* = 0.525 |  | -0.03 | 0.03 | 111 | -1.01 | *p* = 0.914 |
| 60-70 – 70-80 | 25.9 | 18.8 | 111 | 1.4 | *p* = 0.734 |  | 0.05 | 0.03 | 111 | 1.7 | *p* = 0.534 |

Appendix 4. Main effects and interactions of the main models when cut in two parts to avoid an absence of effect due to the linear fitting to a quadratic distribution. Since two models were necessary to test the same hypothesis, according to the Bonferroni correction, the significance threshold equals 0.025.

| Model: *lmer(log(RT) ~ Previous.trial*Current.trial*Age.groups + Presentation.order + (1+Current.trial\|Subjects) + (1\|Items), data = data, REML = FALSE)* | | | | | | | |
| --- | --- | --- | --- | --- | --- | --- | --- |
|  | Model from children to young adults | | |  | Model from young adults to elderly | | |
| Main effects and interactions | F | df | *p*-value |  | F | df | *p-value* |
| Current trial condition | 107.34 | 2, 33.93 | *p* < 0.001* |  | 206.63 | 2, 40.13 | *p* < 0.001* |
| Previous trial condition | 15.93 | 2, 2834.93 | *p* < 0.001* |  | 11.15 | 2, 2713.06 | *p* < 0.001* |
| Age groups | 21.02 | 2, 57.54 | *p* < 0.001* |  | 14.79 | 3, 73.31 | *p* < 0.001* |
| Presentation order | 5.94 | 1, 3074.8 | p = 0.015* |  | 0.04 | 1, 2998.34 | *p* = 0.848 |
| Previous * current trial condition | 7.46 | 4, 2830.28 | *p* < 0.001* |  | 3.23 | 4, 2634.63 | *p* = 0.012* |
| Current trial condition * age-groups | 1.01 | 4, 68.97 | *p* = 0.409 |  | 2.43 | 6, 78.63 | *p* = 0.033(*) |
| Previous items condition * age-groups | 1.86 | 4, 2835.12 | *p* = 0.115 |  | 0.94 | 6, 2497.4 | *p* = 0.466 |
| Previous * current items condition * age groups | 0.79 | 8, 2845.68 | *p* = 0.608 |  | 1.58 | 12, 2454.39 | *p = 0.091* |

Appendix 5. Percentages of rejected trials regarding the SCE conditions on the young adults sample and over the entire lifespan

|  | 10 – 13 | 16 – 18 | 20 – 30 | Young adults (Study 1) | 40 – 50 | 60 – 70 | 70 – 80 | Lifespan |
| --- | --- | --- | --- | --- | --- | --- | --- | --- |
| CC | 7.5% | 3.3% | 2.71% | 2.78% | 3.29% | 3.01% | 3.12% | 3.85% |
| CI | 27.86% | 22.5% | 12.5% | 14.02% | 11.28% | 16.27% | 18.93% | 18.32% |
| CN | 6.14% | 4.55% | 3.18% | 3.2% | 2.63% | 1.01% | 2.05% | 3.3% |
| IC | 10.36% | 4.29% | 6.79% | 3.97% | 3.76% | 3.97% | 3.21% | 5.43% |
| II | 23.8% | 17.2% | 17% | 20.3% | 11.58% | 15.33% | 19.8% | 17.54% |
| IN | 7.38% | 2.86% | 3.1% | 2.65% | 2.01% | 1.06% | 3.10% | 3.3% |
| NC | 6.59% | 5.23% | 5.91% | 4.88% | 3.11% | 2.02% | 2.27% | 4.23% |
| NI | 22.38% | 14.29% | 13.81% | 15.17% | 10.28% | 15.61% | 12.38% | 14.81% |
| NN | 7.19% | 3.12% | 2.81% | 3.01% | 3.62% | 1.04% | 1.56% | 3.26% |
